# Efficacy of Tolvaptan in Older Adults Undergoing Cardiac Surgery: A Single-Center Retrospective Analysis

**DOI:** 10.21470/1678-9741-2023-0507

**Published:** 2024-11-18

**Authors:** Lin Chen, Min Zhou, Dingliang Lv, Shuiwei Qiu

**Affiliations:** 1 Department of Cardiothoracic Surgery, The Quzhou Affiliated Hospital of Wenzhou Medical University, Quzhou People's Hospital, Quzhou, People’s Republic of China

**Keywords:** Tolvaptan, Diuretics, Length of Stay, Cardiac Surgical Procedures, Aged, Kidney, Potassium, Sodium

## Abstract

**Introduction:**

Globally, cardiovascular diseases remain a predominant cause of mortality. Effective fluid management is particularly critical in older adults undergoing cardiac surgery, due to their heightened risk of postoperative complications. Tolvaptan, an oral vasopressin V2 receptor antagonist, has emerged as a promising agent for fluid regulation in cardiac patients. However, its efficacy in the elderly undergoing cardiac surgery is not thoroughly evaluated.

**Methods:**

This single-center retrospective analysis included 146 older adults (≥ 65 years) who underwent cardiac surgery between January 2018 and December 2022. Patients were categorized into two groups: those receiving tolvaptan and a control group receiving traditional diuretics post-surgery. We assessed several outcomes, including hospital length of stay, 30-day mortality, postoperative renal function, and complications.

**Results:**

The study found a significantly reduced hospitalization duration in the tolvaptan group (P=0.044), with no escalation in adverse events. The tolvaptan cohort exhibited a considerable increase in urine output on the postoperative day (POD) three (P=0.003), indicating enhanced renal function and fluid management. Serum creatinine levels notably declined by POD3 (P=0.012), and blood urea nitrogen levels were appreciably lower by POD5 (P<0.001) in the tolvaptan group. Furthermore, serum sodium levels significantly escalated on POD3 and POD5 (P<0.01) in this group, while serum potassium levels remained unchanged.

**Conclusion:**

Tolvaptan significantly optimizes postoperative fluid management in older adults undergoing cardiac surgery. Its administration is linked to improved renal function and a shortened hospital stay, without amplifying adverse effects. These insights could enhance clinical practices and facilitate the management of fluid overload in this vulnerable demographic.

## INTRODUCTION

**Table:** 

Abbreviations, Acronyms & Symbols				
AAR	= Aortic arch replacement		eGFR	= Estimated glomerular filtration rate
AVR	= Aortic valve replacement		MVP	= Mitral valve plasty
BMI	= Body mass index		MVR	= Mitral valve replacement
BUN	= Blood urea nitrogen		NYHA	= New York Heart Association
CABG	= Coronary artery bypass grafting		PAP	= Pulmonary arterial pressure
CI	= Cardiac index		PCWP	= Pulmonary capillary wedge pressure
COPD	= Chronic obstructive pulmonary disease		POD	= Postoperative day
CVD	= Cardiovascular diseases		SBP	= Systolic blood pressure
CVP	= Central venous pressure		TAR	= Total aortic replacement
DBP	= Diastolic blood pressure			

Cardiovascular diseases (CVD) rank among the foremost causes of mortality globally, posing a significant threat to human health^[1]^. Epidemiological studies report over 17 million CVD-related deaths annually, representing nearly one-third of all global mortalities^[2]^. The primary contributors to these deaths are coronary heart disease and stroke, often linked to lifestyle factors like smoking, unhealthy eating habits, inadequate physical activity, and excessive alcohol consumption^[3-5]^. While pharmacological treatments and lifestyle changes are vital, surgical interventions, including both interventional and open-heart surgeries, are pivotal in CVD management. However, these procedures pose significant risks, including perioperative complications such as atrial fibrillation, bleeding, and hypotension^[6]^, which markedly increase mortality risks in these patients.

Vasopressin, an endogenous antidiuretic hormone, plays a key role in modulating vascular tone through its vasoconstrictive effects^[7]^. Emerging research highlights vasopressin's potential in reducing atrial fibrillation post-cardiac surgery compared to traditional agents like epinephrine^[8]^. For instance, a randomized controlled trial by Papadopoulos et al.^[9]^ revealed that low-dose vasopressin prophylaxis improves postoperative hemodynamics in coronary artery disease patients and reduces the incidence of vasoplegic shock, particularly in patients with compromised ejection fractions. Notably, the V1 and V2 receptors of vasopressin exhibit significant differences in structure, localization, and physiological function. While V1 receptors are predominantly found in vascular and central nervous systems, V2 receptors are primarily located in the kidneys. V1 receptors are mainly found in vascular and central nervous systems and influence vasoconstriction and hormone secretion^[10]^. In contrast, V2 receptors, located primarily in the kidneys, are crucial for renal water reabsorption and maintaining internal water and electrolyte balance, especially during cardiac surgery^[11,12]^. Disruptions in this balance can lead to complications like hyponatremia, especially when diuretics are used or in the context of heart failure.

Tolvaptan, a selective oral vasopressin V2 receptor antagonist, has demonstrated efficacy in increasing serum sodium levels in heart failure patients^[13]^. It has been shown to effectively alleviate organ congestion in cardiac surgery patients, enhancing recovery without causing hemodynamic disturbances, electrolyte imbalances, or renal dysfunction. This, in turn, can significantly reduce hospital stay durations^[14]^. However, research by Kiuchi et al.^[15]^ showed that, in contrast to prolonged use, early administration of tolvaptan may extend the discharge time in elderly patients with heart failure.

In light of these findings, our study aims to explore the effectiveness of tolvaptan in the perioperative management of elderly patients undergoing major cardiac surgery. By analyzing medication and monitoring data, we seek to provide a deeper understanding of tolvaptan's role in this demographic, thereby enhancing our knowledge of its potential in improving postoperative outcomes in elderly cardiac surgery patients.

## METHODS

### Study Design and Patients

This study included elderly patients who underwent cardiac surgery in our hospital between January 2018 and December 2022. The inclusion criteria were: (1) age ≥ 65 years old; and (2) having undergone cardiac surgery. Exclusion criteria were: (1) patients with severe preoperative renal dysfunction; (2) patients with advanced heart failure; (3) lack of complete clinical data or observation indicators; (4) patients with hemodynamic instability; and (5) patients with past adverse reactions to tolvaptan or traditional diuretics. Patients were divided into tolvaptan group and control group according to the use of tolvaptan in the perioperative period. This study was approved by the Ethics Committee of Quzhou People's Hospital (AF/SW-05/01.1&2023-086).

### Data Collection

Baseline characteristics were collected from electronic medical records, including sex, age, body mass index (BMI), comorbidities, preoperative renal function indicators and hemodynamic indicators, preoperative New York Heart Association (NYHA) classification, and surgical method. In addition, patient weight, hospital length of stay, urine output, serum creatinine, blood urea nitrogen (BUN), serum sodium and potassium levels, mortality, duration of ventilator support, blood loss from reoperation, length of readmission within 30 days, and postoperative complications were collected.

### Outcome Measures

Primary outcome measures included hospital length of stay and 30-day mortality. Length of hospitalization was measured from the day of surgery to discharge. Mortality rate is the proportion of hospitalized patients who died during hospitalization.

Secondary outcome measures included ventilator support beyond 48 hours, bleeding requiring reoperation, readmission within 30 days, incidence of complications, and postoperative renal function parameters. Daily urine output is the total amount excreted in 24 hours. Electrolyte levels were obtained from blood tests, serum creatinine and BUN were obtained from renal function tests, and complication rates were obtained from the proportion of patients who experienced any adverse event during hospitalization.

### Statistical Analysis

Statistical analysis was performed using IBM Corp. Released 2013, IBM SPSS Statistics for Windows, version 22.0, Armonk, NY: IBM Corp. Graphpad Prims 9.5 was used for graphing. Baseline patient characteristics were analyzed descriptively, with normal distribution expressed as mean and standard deviation. Continuous variables between groups were compared using Student's *t*-test or Mann-Whitney U test, while categorical variables were compared using the chi-square test or Fisher's exact test. Repeated measures analysis of variance was used to analyze data over time. A *P*-value < 0.05 was considered statistically significant.

## RESULTS

### Baseline Characteristics

**Fig. 1:**
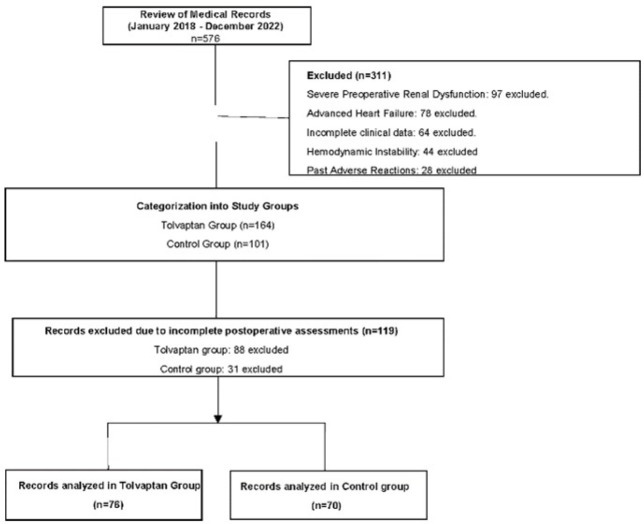
Flow diagram of the included patients.

A total of 146 older adults undergoing cardiac surgery was evaluated in this study, among which 76 receiving tolvaptan while 70 receiving traditional diuretics after surgery (Figure 1). Both groups were comparable in terms of demographic and clinical characteristics, including age, sex, BMI, comorbidities, preoperative NYHA class, and type of surgery. Besides, preoperative indicators related to renal function and hemodynamics also had no significant differences between the two groups (Table 1).

**Table 1:** Baseline characteristics in tolvaptan and control groups.

Characteristics	Tolvaptan Group (n=76)	Control Group (n=70)	*P*-value
Age (years)	68.54 ± 9.34	69.21 ± 8.76	0.512
Sex (female), n (%)	30 (39.47%)	33 (47.14%)	0.350
BMI	25.14 ± 2.63	25.77 ± 3.56	0.217
Comorbidities, n (%)			
Hypertension	45 (59.21%)	42 (60.00%)	0.923
Diabetes	16 (21.05%)	18 (25.71%)	0.506
Hyperlipidemia	34 (44.74%)	32 (45.71%)	0.906
COPD	22 (28.95%)	24 (34.29%)	0.488
Cerebral infarction	11 (14.47%)	13 (18.57%)	0.505
NYHA class III-IV, n (%)	8 (10.53%)	9 (12.86%)	0.661
Preoperative renal function			
Creatinine (mg/dL)	1.02 ± 0.41	1.04 ± 0.38	0.742
BUN (mg/dL)	17.54 ± 5.31	18.21 ± 6.14	0.541
eGFR (mL/min/1.73 m^2^)	67.35 ± 20.32	65.79 ± 19.68	0.620
Preoperative hemodynamics			
Heart rate (beats/min)	72.32 ± 13.35	71.83 ± 14.13	0.831
SBP (mmHg)	126.74 ± 16.53	125.27 ± 15.94	0.642
DBP (mmHg)	78.54 ± 12.24	77.13 ± 11.68	0.554
PAP (mmHg)	36.18 ± 10.53	35.62 ± 10.82	0.743
PCWP (mmHg)	15.26 ± 6.42	15.68 ± 6.94	0.708
CVP (mmHg)	10.15 ± 3.94	9.84 ± 4.12	0.641
CI (L·min⁻^1^·m⁻^2^)	2.91 ± 0.63	2.82 ± 0.57	0.560
Surgical procedure, n (%)			0.787
AVR	29 (38.16%)	32 (45.71%)	
MVR	5 (6.58%)	5 (7.14%)	
MVP	19 (25.00%)	14 (20.00%)	
CABG	15 (19.74%)	10 (14.29%)	
AAR or TAR	8 (10.53%)	9 (12.86%)	

AAR=aortic arch replacement; AVR=aortic valve replacement; BMI=body mass index; BUN=blood urea nitrogen; CABG=coronary artery bypass grafting; CI=cardiac index; COPD=chronic obstructive pulmonary disease; CVP=central venous pressure; DBP=diastolic blood pressure; eGFR=estimated glomerular filtration rate; MVP=mitral valve plasty; MVR=mitral valve replacement; NYHA=New York Heart Association; PAP=pulmonary arterial pressure; PCWP=pulmonary capillary wedge pressure; SBP=systolic blood pressure; TAR=total aortic replacement

### Postoperative Clinical Outcomes

As shown in Table 2, the length of hospital stay was significantly shorter in the tolvaptan group compared to the control group (*P*=0.044). There were no significant differences in 30-day mortality, ventilator support over 48 hours, bleeding requiring reoperation, and rehospitalization within 30 days. The incidence of complications was similar between the two groups, including acute kidney injury, atrial fibrillation, wound infection, stroke, and myocardial infarction.

**Table 2:** Postoperative clinical outcomes in tolvaptan and control groups.

	Tolvaptan Group (n=76)	Control Group (n=70)	*P*-value
Length of hospital stay (days)	6.95 ± 2.14	7.82 ± 2.36	0.044^*^
30-day mortality, n (%)	0 (0%)	1 (1.43%)	0.479
Ventilator support > 48 h, n (%)	6 (7.89%)	8 (11.43%)	0.469
Bleeding requiring reoperation, n (%)	4 (5.26%)	5 (7.14%)	0.738
Rehospitalization within 30 days, n (%)	5 (6.58%)	6 (8.57%)	0.649
Complications, n (%)			
Acute kidney injury	9 (11.84%)	13 (18.57%)	0.256
Atrial fibrillation	17 (22.37%)	21 (30.00%)	0.294
Wound infection	2 (2.63%)	3 (4.29%)	0.671
Stroke	1 (1.32%)	2 (2.86%)	0.607
Myocardial infarction	2 (2.63%)	3 (4.29%)	0.671
^*^P<0.05			

### Postoperative Renal Function

**Fig. 2:**
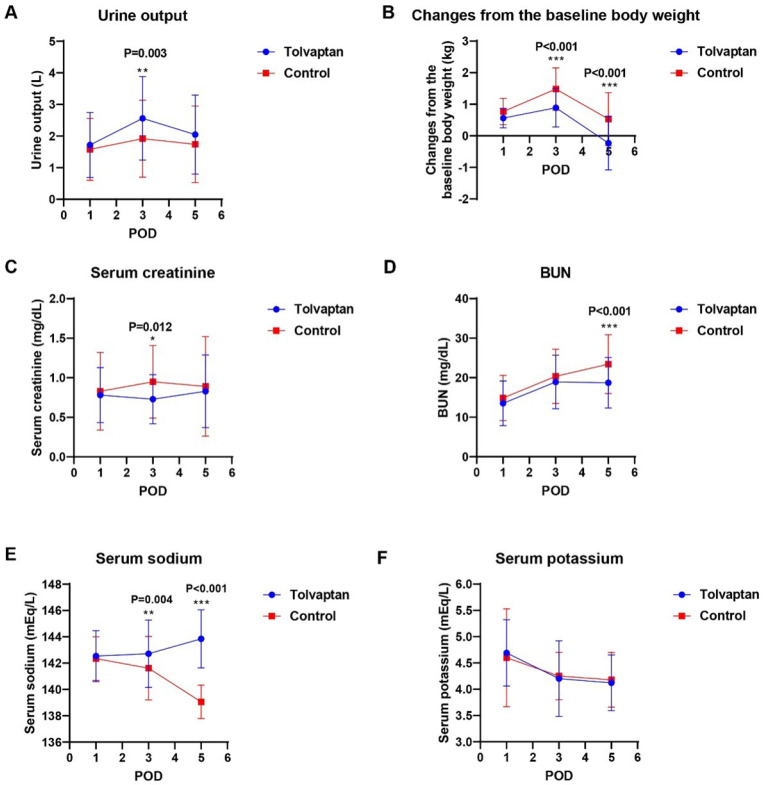
Postoperative renal function in tolvaptan and control groups. (A) Daily urine output; (B) changes from the baseline body weight; (C) serum creatinine levels; (D) blood urea nitrogen (BUN) levels; (E) serum sodium levels; (F) serum potassium levels. *P<0.05, **P<0.01; ***P<0.001, compared with the control group. POD=postoperative day.

Analysis of postoperative renal function parameters revealed a significant increase in urine output on postoperative day (POD) three in the tolvaptan group but not in the control group (*P*=0.003), while the urine output on POD5 was similar in both groups (Figure 2A). Compared to the control group, the baseline weight change was smaller in the tolvaptan group, both in POD3 and POD5 (both *P*<0.001) (Figure 2B). In addition, serum creatinine levels showed a significant decrease on POD3 (*P*=0.012) (Figure 2C), and BUN was significantly lower on POD5 in the tolvaptan group compared to the control group (*P*<0.001) (Figure 2D). However, serum sodium levels significantly increased in the tolvaptan group both on POD3 and POD5 (*P*<0.01) (Figure 2E), but serum potassium levels showed no significant difference compared to the control group (Figure 2F).

## DISCUSSION

Due to the decline of physiological functions, especially heart and kidney function, older adults are prone to fluid disorders such as volume overload or dehydration, making their fluid management particularly critical after cardiac surgery^[16,17]^. This susceptibility underscores the need for effective fluid management strategies to mitigate postoperative complications like acute kidney injury and heart failure, which significantly impact patient recovery and prognosis^[16,18]^.

In cardiac surgery, particularly for the elderly, maintaining adequate urine output is crucial for ensuring renal perfusion and preventing acute kidney injury^[19]^. Tolvaptan, by inhibiting antidiuretic hormone receptors, reduces water reabsorption in renal tubules^[20]^. In this study, the diuretic effect of tolvaptan, evidenced by higher urine output, suggested improved renal function and fluid balance. Lesser changes in baseline body weight in the tolvaptan group also indicated effective management of fluid retention.

Renal dysfunction is a common complication after cardiac surgery, and elderly patients are more likely to experience renal insufficiency after surgery^[21]^. In addition to affecting heart function, renal function impairment can also increase hospital stay and treatment costs, even related to the increase of long-term mortality^[19,22]^. Therefore, paying attention to changes in renal function is a key aspect of postoperative care. In this study, the significant decrease in serum creatinine levels at POD3 and the decrease in BUN levels at POD5 in the tolvaptan group suggested that tolvaptan might help alleviate postoperative renal burden, thereby reducing the risk of acute kidney injury. This was consistent with previous studies, indicating that tolvaptan can improve renal function without adverse effects on other clinical outcomes^[23-25]^. In addition, the kidney is the main organ that maintains electrolyte balance, so electrolyte abnormalities can indicate impaired kidney function^[26]^. As is well known, tolvaptan can lead to serum sodium increase^[27]^, which was consistent with our results. Therefore, in older adults, the use of tolvaptan requires careful monitoring of serum sodium levels to avoid excessive sodium retention and potential hypernatremia, especially for elderly cardiac surgery patients who already have a tendency towards hypernatremia or a risk of electrolyte imbalance. Potassium is a key electrolyte for cardiac function, and fluctuations in its level may affect the electrophysiological stability of the heart^[28]^. The research results showed that there was no significant difference in serum potassium levels between the tolvaptan group and the control group, suggesting that while increasing urine output, tolvaptan does not significantly affect potassium excretion.

### Limitations

Nonetheless, there are still some limitations in this study. The retrospective design and the single-center nature of the study might limit the generalizability of the results. Additionally, the small sample size may not adequately represent the broader elderly population undergoing cardiac surgery. Future studies should aim to include a larger, more diverse population and possibly a multi-center design to validate our findings.

In summary, the results suggest that tolvaptan has a favorable impact on fluid management, renal function, and overall recovery in elderly cardiac surgery patients. This is particularly relevant given the increased risk of fluid and electrolyte imbalances, renal dysfunction, and prolonged hospitalization in this age group. Tolvaptan's role in enhancing urine output and minimizing fluid retention without significantly altering serum potassium levels is a valuable therapeutic advantage.

## CONCLUSION

Tolvaptan appears to offer significant benefit in managing postoperative fluid balance in older adults undergoing cardiac surgery. Its use is associated with improved renal function and reduced hospital stay without an increase in adverse outcomes. These findings can inform clinical practice and guide the management of fluid overload in this high-risk patient population.
